# The path to Net Zero carbon emissions for veterinary practice

**DOI:** 10.3389/fvets.2023.1240765

**Published:** 2023-11-06

**Authors:** Jeremy A. Watson, Corinna Klupiec, Jane Bindloss, Mariane Morin

**Affiliations:** ^1^Brimbank Veterinary Clinic, Melbourne, VIC, Australia; ^2^Metamorphomedia, Sydney, NSW, Australia; ^3^SANE Veterinary Management, Melbourne, VIC, Australia; ^4^Thornbury Veterinary Hospital, Melbourne, VIC, Australia

**Keywords:** veterinary, practice, carbon, emissions, zero, climate, change

## Abstract

The urgent need to reduce greenhouse gas emissions in line with the Paris Agreement is a compelling reason for the entire veterinary profession to act on climate change because of its impact on animal health. The aim of this perspective is to provide a business framework that veterinary practices can use to implement the path to Net Zero carbon emissions. Practice management is identified as a key stakeholder capable of implementing significant change within the sector. Climate related business opportunities and challenges are identified and integrated into a stepwise process for practices to follow. The pathway requires establishing a culture of sustainability within the veterinary team, measuring and reporting emissions, setting targets and systematically prioritizing reductions. Practices can begin this process immediately by reducing emissions under direct control of the business (Scope 1) and emissions from electricity purchases (Scope 2). To complete the pathway, emissions from all other activities (Scope 3) will need to be reduced and offset. Reduction of Scope 3 emissions is more challenging and will require collaboration between all supply chain stakeholders. The progression of climate change is now inevitable and a proactive approach from veterinary leaders, in particular practice management, will provide new opportunities, manage risks and inspire the broader veterinary sector to join their efforts to achieve a better future for animal health.

## 1. Introduction

Climate change is having a significant and continuing impact on animal health (1–7). It is now well established that climate change is caused by human activity and therefore everyone, including the veterinary sector, has an important role to play in responding to this situation (8, 9). Mitigation of climate change and its impacts requires maximal reduction of anthropogenic greenhouse gas emissions. For residual emissions that cannot be avoided, offsetting can be used so that the net impact on climate change is zero. This is known as Net Zero emissions, commonly shortened to Net Zero. In this perspective, we propose a framework for veterinary businesses to implement a pathway to Net Zero carbon emissions. Our focus is on small to medium sized veterinary practices; however, the principles can be applied to businesses of any size. Integrated action by all veterinary businesses will be crucial for accelerating decarbonization of the veterinary sector in line within globally accepted emissions reduction timelines.

The Paris Agreement aims to keep global warming to 1.5°C, or as near as possible, above pre-industrial levels (10). The window of opportunity to meet the 1.5°C target is rapidly closing; to accomplish this goal, global greenhouse gas emissions must be reduced by 45% by 2030 (from 2010 levels), and Net Zero emissions must be achieved by 2050 (10, 11). However, the current trajectory is for greater than 2°C of global warming (10). The difference between 1.5°C and 2°C will have a significantly greater impact on animal health and society in general (10, 12).

The veterinary sector has an important role to educate and inspire the community to deal with this issue (13). When compared to the broader non-science trained community, the background of veterinarians provides them with an advantage in being able to analyze, comprehend and communicate climate science (14). The community looks to veterinarians to provide leadership on issues of animal health which should include education about the impact of climate change. Research indicates veterinarians understand that human induced climate change is occurring, and there is desire to take action in their professional lives, but this has not resulted in corresponding change within the businesses in which veterinarians work (13–17). To drive implementation of a path to Net Zero within the veterinary sector, veterinary practice management needs to combine the imperative of climate science with the opportunities and challenges it presents and align this with existing business management structures, opportunities and obstacles.

There is published work providing examples of common opportunities for emissions reduction in veterinary practice (18, 19). To drive impactful deployment of such activities throughout the sector, key business decision makers need to be identified and provided with details of how to implement a pathway to Net Zero. Within veterinary practices, the business decision makers are the owners and managers (management). They are the key stakeholders who allocate resources to implement the changes needed. When analyzing prospective changes, management will apply the fundamental business considerations of profitability, team purpose and client value. The inevitable progression of climate change will result in external pressures and internal opportunities requiring successful business responses with due consideration to all three of these factors (20).

External pressures include:

- Growing community expectations (14)
- Attracting, motivating and retaining talented employees, particularly younger individuals who place value on practices demonstrating a clear commitment to their future (21, 22)
- Preparing for future compliance with government regulation and industry policy (23–26)
- Responding to climate related business risks. The Task Force on Climate-Related Financial Disclosure is widely used in corporate risk reporting and can be applied to a business of any size (27). Climate risk is broken into:
  a) Physical risks such as extreme weather events impacting business, staff, clients and patients particularly in practices located in areas vulnerable to flooding or wildfire.
  b) Transitional risks: equipment and processes will become redundant in the transition to low carbon alternatives. For example, gas heating will be replaced by electric heat pump alternatives and anesthetic gases will need a zero-emissions alternative (28–32)
  c) Litigation risks: Especially for corporate entities, litigation risk may be present in the event of a failure to disclose climate risks to shareholders.

Internal opportunities include:

- Financial efficiency savings from supply chain review and implementation of new green technology (20)
- Alignment of caring for animal health with existing practice culture
- Increased client and staff loyalty by marketing businesses’ climate change commitments (20, 33)
- Business growth by developing advice on emissions reduction in production animals. Improved animal health reduces emissions and improves productivity for producers (5, 34–36)

Considering these factors, the existing interest among veterinary teams to do more to address climate change is likely to increase. To drive meaningful progress towards Net Zero, it is important that veterinary managers are equipped to make informed decisions and allocate the necessary resources within an appropriate framework (18). Practices will proceed down this path if they are aware of opportunities, how they can be exploited and are provided with solutions to obstacles they may encounter.

**Figure 1:**
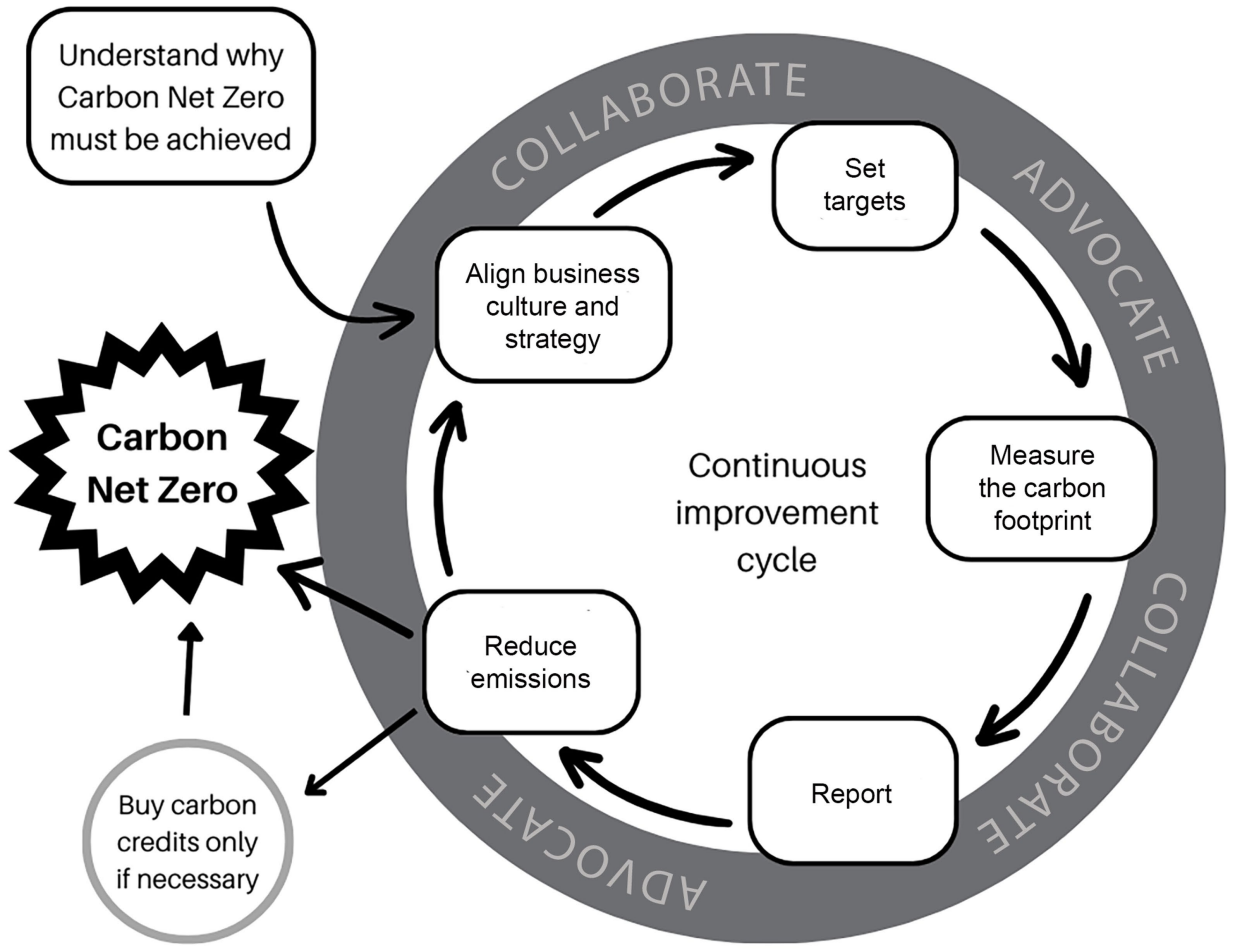
The path to Net Zero highlighting the importance of continuous collaboration, evaluation and improvement.

Emissions reduction by business is now well developed in the corporate non-veterinary economy and provides guidance for developing a pathway for the veterinary sector. In this perspective we draw on this model to provide a framework for veterinary managers to follow (Figure 1). It begins with recognising the need for change and then implementing change within a well-defined pathway. Conceptually this perspective draws on “Targeting Net Zero – a strategic framework for business action” and applies this to the veterinary context (37).

## 2. The pathway to Net Zero for veterinary practice

### 2.1. Aligning business culture and strategy

Setting a culture of sustainability within the practice is a critically important foundation for a successful Net Zero strategy. In a busy practice, there are many daily touch points where the veterinary team needs to make routine decisions based on established criteria; for example, point of use recycling, anesthetic flow rates and procurement options. Therefore, it is important that climate-related goals become deeply embedded in the workplace culture. This change in mindset also needs to align with the existing strategy and purpose of the practice to avoid conflict with current budgets, timelines, stakeholders and targets (38).

In small to medium sized veterinary practices, the workplace culture is influenced by the daily exposure of the team to the actions and attitudes of the senior practice principal(s) or onsite manager(s). As key stakeholders, these individuals must appreciate that a successful Net Zero strategy requires cultural change within the practice. Importantly, all team members need to understand climate change and believe their actions will contribute to reducing emissions. Tools that can assist with achieving these outcomes include free online materials, customized programs and external consultants (39–41).

### 2.2. Setting targets

Once a practice has made the decision to implement a pathway to Net Zero it is important to set targets based on scientific evidence. Annual emissions reduction targets should be set in accordance with a near term (5–10 year) target and the final target should be Net Zero by 2050. The Science Based Targets Initiative is an international standard which aligns with the 1.5°C pathway (Paris Agreement) and sets out key requirements for businesses to establish validated targets (42). It recommends setting a near term target of 50% reduction by 2030 and 90% reduction by 2050, with residual emissions neutralized with carbon credits.

### 2.3. Measuring the carbon footprint

To calculate the carbon footprint, all emissions occurring as a result of the activities of the business must be identified and quantified (19). This can be facilitated by using published recommendations, customized carbon calculators, or engagement of an external consultant (19, 43). Free online carbon calculators can also provide a useful starting point for some types of emissions (44).

When calculating emissions, it is important to define the boundary between what is, and is not, included. For example, a can of pet food consumed by a patient within the practice may be part of the footprint of the practice, but a can of pet food sold by the business and consumed offsite by the customer’s pet may not. Comprehensive information about setting emissions boundaries is detailed in the Green House Gas Protocol (45).

It is also important to consider why the carbon footprint is being calculated as this will influence the level of accuracy and detail of the results. During the measurement process, the business also needs to consider how the results will be reported (see section 2.4) and to whom. For example, reporting to a public register for the purposes of purchasing carbon offsets may be different in detail to internal reporting to the veterinary team for the purposes of emissions reduction. Furthermore, the process of calculating needs to be transparent so that year on year comparisons are meaningful as the business grows or if the methodology of calculation changes.

Emissions are divided into three categories (see also Table 1):

**Table 1:** Examples of the three scopes of emissions commonly encountered in veterinary businesses.

Emission boundary	Common examples in veterinary business
Scope 1	Gas/oil heating, combustion engine vehicles owned and used in the business, anesthetic gas and escaped refrigeration gas
Scope 2	Electricity purchased from the grid
Scope 3	Waste disposal, water, employee commute, animal cremation, conference travel and expenses, in-house catering, medical consumables and chemicals, external consultants, delivery of supplies

Scope 1 – emissions produced onsite.

Scope 2 – emissions produced from purchased electricity.

Scope 3 – emissions that are triggered by operation of the business due to purchase and use of products and services.

Published emission factors are used to convert usage data from each scope to an equivalent tons of CO_2_ emissions (CO_2_e) (19, 46). Calculation of emissions factors is known as Life Cycle Analysis (LCA) and is a technical process involving “cradle-to-grave” carbon analysis which is usually financed and undertaken by the manufacturer (46). In the absence of LCA values, an estimate of the carbon footprint of these items is determined using the purchase price which is then converted to a CO_2_ equivalent using internationally accepted accounting factors (economic input–output LCA) (47, 48).

A key recommendation of this perspective is to begin by measuring Scope 1 and 2 emissions. This data is easy to collect and there is direct control over emissions (19). For example, data is sourced from energy bills, vehicle odometer readings, maintenance reports and purchasing invoices. All veterinary practices can commence their path to Net Zero immediately by measuring usage of these items year on year and choosing lower carbon alternatives. This initial step does not require the use of carbon calculators or consultants to convert usage data into CO_2_ equivalent.

Entire carbon footprint measurement is more complex and requires inclusion of data for Scope 3 emissions, as these are likely to be the largest component of the three scopes (19). Measurement of some Scope 3 emissions is quite straightforward (e.g., employee commute, landfill waste volumes and water use), however, to be complete, medical consumables, chemicals, reagents and other items need to be included. For many of these, there are no published emissions factors, though data from the human health sector may be available (47). More emphasis on LCA by suppliers is required to equip veterinary practices to measure Scope 3 emissions accurately (49). Due to the complexity of data collection and emissions conversion factors, using a carbon auditing consultant is recommended to calculate the entire carbon footprint of a practice.

### 2.4. Reporting

Reporting can be as simple as notifying internal management and the veterinary team to identify areas for reduction. More advanced public reporting either via the practice’s communication channels or via a public register enables amplification of the value of achievements to the client base. There are numerous public registers (45). Registries may be administered by governments, non-government organizations (NGOs) or industry groups and may provide advice on emissions reductions. Reporting to other stakeholders in the value chain is increasingly important (45).

As a minimum, Scope 1 and 2 emissions should be reported (19). If only Scope 1 and 2 emissions are measured, it must be clear in any public reporting that Scope 3 emissions are excluded. When Scope 3 emissions are included, calculation of the total carbon footprint to an acceptable international standard, suitable for reporting on a reputable public register, is a more complex task (45, 50). Using the services of a specialist consultant is recommended as process integrity is important to avoid a challenge of making false advertising claims, also known as “greenwashing”.

### 2.5. Reducing emissions

Implementing reduction strategies can be prioritized once emission sources are understood. Specific veterinary emissions reduction strategies have been published by several authors (19, 29, 40, 51). Scope 1 and 2 emissions should be the priority for reduction given they are easily measurable and are under direct control of the business. Many of these emissions have zero-emission options available, often with cost efficiency gains. One significant industry specific challenge for Scope 1 reduction in the veterinary sector is to achieve a zero-emission alternative to current anesthetic gas use (16, 28–30, 52).

Scope 3 emissions have some existing solutions, for example employees could commute by electric vehicles powered by renewable electricity. Other more complex specific veterinary/medical items such as disposable surgical items, chemical reagents and single use hospital consumables will require further research and technological advances to find zero carbon alternatives. While reduction of Scope 3 emissions is a crucial component of achieving Net Zero within the veterinary sector, “greening” of the supply chain goes beyond the responsibility of the end user and will require collaboration between all stakeholders, including manufacturers and distributors. Models for working with suppliers on integrated solutions within the human health sector, such as the National Health Service (NHS) supplier roadmap, could be adapted for the veterinary sector (49).

### 2.6. Purchasing carbon credits

For the veterinary sector to achieve Net Zero by 2050 it is likely that some carbon credits will need to be purchased to offset emissions that cannot be eliminated. A carbon credit represents one ton of carbon removed from the atmosphere. It must be permanent (>100 years), additional (i.e., a new or extra process), verifiable, audited and registered on a public register so it can only be used once. Carbon credits play an important role in reducing atmospheric carbon, supporting biodiversity and sustainable agriculture, however the current market may be subject to irregularities and due diligence is recommended before purchasing (53). Once the carbon footprint has been calculated and audited, quality carbon credits can be purchased to neutralise any remaining emissions and reported on a public registry.

### 2.7. Advocating and collaborating

Publicly promoting the benefits of a sustainability program that includes emissions reduction creates client value. Clients are willing to pay more for services from a practice that has demonstrated sustainability achievements (33). Publicizing progress on sustainability provides leadership to colleagues, team members and clients, and encourages others to join and support the efforts of the business. This can create positive feedback loops.

## 3. Discussion

The veterinary profession is becoming increasingly aware that it will be confronted directly by the impacts of climate change, and that it has an obligation to take meaningful action to help safeguard animal health (8, 9, 17–19). Veterinary practice management is a key stakeholder in this process, as it has the power to provide leadership and allocate appropriate resources to a mitigative response. This perspective identifies how emissions reduction can be achieved by embracing a structured pathway to Net Zero. However, this systematic approach represents a significant shift for many veterinary practices and there are several knowledge gaps and obstacles that must be addressed to ensure its successful deployment.

Further research is needed to evaluate the awareness of veterinary management of the rationale for Net Zero, and of the level of motivation to implement emissions reduction strategies. A greater understanding of mechanisms for promoting participation in, and “ownership” of, a Net Zero pathway by all members of the veterinary team would also be beneficial. The resulting information could be used to design education programs to stimulate business engagement. These could be delivered through existing management information channels such as education institutions, symposia, advocacy groups, professional associations, supply company representatives, publications, social media, and conversations between colleagues (13, 54).

Measuring and reporting of emissions are key components of a Net Zero strategy. Time constraints and technical complexity involved in measuring are barriers that could be addressed with the development of software programs that capture and analyze data. This is already occurring in the wider corporate sector (35). Difficulties in measuring Scope 3 items include the lack of data for life cycle analysis of specific medical products. To address this, stakeholders can organize and collectively lobby manufacturers to undertake life cycle analysis and make the data available to the veterinary sector. Veterinary leadership organizations, such as professional and regulatory bodies, could provide stewardship in this process. Moreover, action towards Net Zero needs to be undertaken at all stages of a product’s life, from manufacture to end use, to ensure that the responsibility for emissions reduction is borne appropriately by all relevant parties. Economic modelling that demonstrates cost savings from supply chain review and integration of green technologies would help justify expenditure on the specialist resources needed for accurate Scope 3 carbon auditing.

The long-term challenge with industry specific veterinary/medical emissions is to develop zero carbon alternatives. This will necessitate extensive collaboration. Engaging all stakeholders in the value chain by forming a taskforce is commonly used by government and industry leadership organizations in other sectors. Again, veterinary leadership organizations could coordinate such initiatives (23). A pathway to zero carbon anesthesia could be used as a model that could subsequently be applied to other complex emissions (29).

Generic carbon registries are available to the veterinary sector, however there is scope for veterinary professional organizations and regulators to develop a registration body for veterinary businesses to report their carbon footprint. This could provide Net Zero certification by offering carbon credits that benefit animal health, as well as being a conduit for industry specific emission reduction resources.

The path to Net Zero can be achieved by following the process outlined in this perspective. The first steps are simple, clear and rewarding, however solutions to complex Scope 3 emissions will be more challenging. Motivating the collective energy of the veterinary sector is urgently needed to collaborate and find solutions to help provide a better future for all animals.

## Data availability statement

The original contributions presented in the study are included in the article/supplementary material, further inquiries can be directed to the corresponding author.

## Author contributions

JW organized the concept of the article and wrote the first draft of the manuscript. CK organized the literature search. All authors contributed to the article and approved the submitted version.
